# Micromorphological changes in cardiac tissue of drug-related deaths with emphasis on chronic illicit opioid abuse

**DOI:** 10.1111/add.12106

**Published:** 2013-04-12

**Authors:** Monika H Seltenhammer, Katharina Marchart, Pia Paula, Nicole Kordina, Nikolaus Klupp, Barbara Schneider, Christine Fitzl, Daniele U Risser

**Affiliations:** Department of Forensic Medicine, Medical University ViennaVienna, Austria

**Keywords:** Drug-related deaths, heroin addiction, micromorphological changes, myocard fibrosis, opiates, opioids

## Abstract

**Aims:**

The main intention of this retrospective study was to investigate whether chronic illicit drug abuse, especially the intravenous use of opioids (heroin), could potentially trigger the development of myocardial fibrosis in drug addicts.

**Design:**

A retrospective case–control study was performed using myocardial tissue samples from both drug-related deaths (DRD) with verifiable opioid abuse and non-drug-related deaths in the same age group.

**Setting:**

Department of Forensic Medicine, Medical University of Vienna, Austria (1993–94).

**Participants:**

Myocardial specimens were retrieved from 76 deceased intravenous opioid users and compared to those of 23 deceased non-drug users.

**Measurements:**

Drug quantification was carried out using the enzyme-multiplied immunoassay technique (EMIT), followed by [gas chromatography–mass spectrometry (GC–MS), MAT 112®], and analysed using the Integrator 3390A by Hewlett Packard® and LABCOM.1 computer (MSS-G.G.). The amount of fibrous connective tissue (FCT) in the myocardium was determined by using the morphometric software LUCIA Net version 1.16.2^©^, Laboratory Imaging, with NIS Elements 3.0®.

**Findings:**

Drug analysis revealed that 67.11% were polydrug users and the same proportion was classified as heroin addicts (6-monoacetylmorphine, 6-MAM)—32.89% were users of pure heroin. In 76.32% of DRD cases, codeine was detected. Only 2.63% consumed cocaine. The mean morphine concentrations were 389.03 ng/g in the cerebellum and 275.52 ng/g in the medulla oblongata, respectively. Morphometric analysis exhibited a strong correlation between DRD and myocardial fibrosis. The mean proportion of FCT content in the drug group was 7.6 ± 2.9% (females: 6.30 ± 2.19%; males: 7.91 ± 3.01%) in contrast to 5.2 ± 1.7% (females: 4.45 ± 1.23%; males: 5.50 ± 1.78%) in the control group, indicating a significant difference (*P* = 0.0012), and a significant difference in the amount of FCT between females and males (*P* = 0.0383). There was no significant interaction of age and FCT (*P* = 0.8472).

**Conclusions:**

There is a long-term risk of cardiac dysfunction following chronic illicit drug abuse with opioids as a principal component. Regular cardiological examination of patients receiving substitution treatment with morphine is strongly recommended.

## Introduction

Irrespective of the noticeable decline in the demand for drugs in recent years, the illegal use of drugs remains a global affair which represents an increasing health problem, particularly in people of younger ages. In the World Drug Report of 2011, the United Nations Office on Drugs and Crime (UNODC) estimates that, in 2009, between 149 and 272 million people, or 3.3 and 6.1% of the global population aged 15–64 years, used illicit substances at least once a year. Among all drug-dependent people world-wide, an estimated 12–21 (mid-point: 16.5) million people are chronic opiate addicts. Heroin is the most commonly used opiate, consumed by about three-quarters of global opiate users. In 2009, there were an estimated 12–14 million opiate users world-wide 1. Heroin is also the main opiate used in Europe. Its prevalence is estimated at 0.6% of the population aged 15–64, or between 3.1 and 3.5 million people. In Austria, a small country in central Europe with approximately 8 million inhabitants, the estimated prevalence is 0.41% 1, where the gender distribution is 0.9% males and 0.2% females, as stated by Uhl *et al*. 2. Uhl & Seidler 3 estimated that there are 17 276 opioid users in Austria and 10 953 in Vienna, the Austrian capital, with a population of almost 1.8 million. Similarly, according to the Austrian Federal Institute for Public Health and Klupp *et al*., opiate-related deaths represent as much as 80% of all drug-related deaths (DRD) in Austria 4,5, the majority of which again occur in Vienna.

According to the drug report of the Federal Ministry of the Internal Affairs in 1994 6, since the 1960s the number of DRD in Austria has been risen steadily, peaking in 1994 with a total of 250 officially registered cases. All DRD cases must be reported to the coroner and have to undergo forensic post-mortems by law 5. At the Department of Forensic Medicine in Vienna, in the 1990s heroin overdose was the main cause of DRD 7,8.

During the post-mortems of DRD, heroin abuse victims frequently displayed an unusually high amount of fibrotic tissue in their hearts. These findings led to a subsequent investigation to determine whether long-term opioid abuse might be linked to specific fibrotic alterations in the myocardium which, in turn, would be very important to know in the event that patients receive substitution therapy with morphine (opioid maintenance treatment with substitution drugs, e.g. methadone or buprenorphine, as described by Aeschbach *et al*. 9). Methadone in particular is believed to be responsible for Q-T interval prolongation and torsades de pointes, which may, in the worst case, lead to lethal negative side effects for patients treated for drug addiction with hearts already damaged by opioid abuse, as stated by Stringer *et al*. 10.

Accordingly, the primary objective of this study was to investigate cases in which cardiac pathology was found regularly in young drug (heroin) abusers in order to support the theory that opioid abuse indeed has a profound impact on the cardiovascular system. Due to the fact that, particularly during the 1990s, the co-consumption of opioids with stimulants such as cocaine was somewhat low (about 6% according to Risser *et al*. 8), all the cases used in this study were taken from the years 1993 and 1994 to obtain a sample of opioid users which were as free from stimulants as possible, and to avoid the potential influence of cocaine or other analeptic agents on fibrous connective tissue (FCT) development in the myocardium.

Several publications, such as those by Darke *et al*. 11, Karch 12 and Milroy & Parai 13, reconsider the role played by opiates or opioids in the development of cardiac pathology; however, with inconsistent results 11–13, as there is a lack of reports about fibrosis. Thus the definitive origin of myocardial fibrosis associated with morphine abuse is still to be determined (Dettmeyer *et al*.) 14. Although it has been established by Milroy & Parai 13 and Dettmeyer *et al*. 14, that fibrotic alterations of the myocardium are associated with the abusive use of stimulants (e.g. cocaine and amphetamines), intravenous opiate abuse and its general association with long-term pathological changes in cardiac tissue have already been identified by Dressler & Roberts 15. In addition to right ventricular dilatation, myocyte necrosis and endo- and myocardial fibrosis, infective endocarditis is the most significant example of pathological alterations in cardiac tissue linked to intravenous drug abuse, and there is compelling evidence that an inflammation of any kind has a significant impact on the development of fibrous connective tissue, especially in the myocardium, according to Menda & Gorbach, Rajs & Falconer and Dressler & Roberts 15–17.

The myocardium consists of various cell types: cardiomyocytes, cardiofibroblasts, endothelial cells and smooth muscle cells. Cardiofibroblasts have the highest cell population, while cardiomyocytes form about two-thirds of the myocardial volume, as stated by Camelliti *et al*., referring to the recent review of Fan *et al*. 18,19. According to Manabe *et al*., fibroblasts proliferate particularly fast in the myocardium and secrete excessively large amounts of collagen type I 20. This accumulation of FCT leads to tissue stiffness, thickening and hardening of the ventricle wall, which results ultimately in an impairment of the cardiac contractile function and adversely affects myocardial viscoelasticity, as described by Burlew & Weber and Manabe *et al*. 20,21. Weber suggests that necrotic cells are replaced by FCT due to inflammatory signals 22. Rajs & Falconer discovered that myocardial fibrosis is the direct adaptive response to chronically increased pressure load, i.e. hypertension 16. As stated by Sun & Weber, Manabe *et al*. and Tenhunen *et al*., a broad range of criteria such as different cells, growth factors and peptides play an important pathogenic role in the development of myocardial fibrosis, underlining the complexity of this process 20,23,24.

## Methods

### Case determination

For this purpose, we performed a retrospective case–control study using myocardial tissue samples from both drug-related deaths (DRD), referred to hereafter as ‘opiate group’ irrespective of whether they used opiates or opioids, and non-drug-related deaths in the same age group. Consequently, forensic post-mortem and police reports of all cases of death by heroin overdose between 1 January 1993 and 31 December 1994 were retrieved from the database of the Vienna Department of Forensic Medicine, in close cooperation with the Vienna Police Department. All cases were reviewed by the authors. Deceased people showing primary atherosclerosis or primary alteration of the small intramyocardial vessels were excluded from this study.

Drug-related deaths have been defined according to edicts from the Austrian Federal Ministry of the Interior and the European Monitoring Centre for Drugs and Drug Addiction (EMCDDA), respectively 25,26. All cases presented in this study underwent a standardized forensic autopsy with examination of all major organs, including microscopy of representative tissue samples (heart, lung, liver, kidney and brain with one myocardial sample per case regularly being taken from the left ventricular myocardium and one pulmonary sample per case from the right inferior lobe). For a quantitative toxicological analysis performed by gas chromatography–mass spectrometry (GC–MS) (Vycudilik 27), both brain tissue and peripheral blood samples are usually collected. Due to the fact that this study was a retrospective analysis, it was based only on tissue samples taken on a regular basis. As such, the autopsies reported here were not collected specifically for this study, but were rather standard forensic post-mortems conducted as part of the legal obligation of the Vienna Department of Forensic Medicine.

The opiate group consisted of a total of 76 cases (63 males, 13 females). In this group, the age ranged from 15 to 38 years with a mean age of 24.9 ± 5.8 years (females: 25.4 ± 5.9; males: 22.6 ± 5.1). All cases in this group were identified as illicit drug users, where death was imputed to morphine/heroin toxicity. Only one individual in the opiate group died of carbon monoxide poisoning, albeit under the influence of heroin.

The control group consisted of 23 cases (16 males, seven females) in which cause of death was according to police reports, not drug-related, e.g. hanging (suicide), carbon monoxide poisoning (accident, suicide without drug influence), stabbing (murder) or polytrauma as a result of road traffic accidents. In this group, ages ranged from 15 to 41 years, with a mean age of 28.4 ± 8.2 years (females: 31.4 ± 8.2; males: 27.1 ± 7.9) (Table 1).

**Table 1:** Summary of morphometric results, age distribution, gender distribution and cause of death with *P*-values

	Opiate group	Control group	Total
Number *n*	76	23	99
Sex			
Male	63	16	79
Female	13	7	20
Age in years (mean)	24.9	28.4	25.7
Cause of death			
Opioid intoxication	75	0	75
Suicide	0	10	10
Polytrauma	1	11	12
CO intoxication	0	2	2
FCT general (*P* = 0.0012)			
Mean %	7.55	5.18	6.37
FCT gender-related (*P* = 0.383)			
Mean % female	6.23	4.45	5.34
Mean % male	7.91	5.50	6.71
Myocardial tissue, mean % (*P* = 0.2349)	79.66	81.45	80.56

CO = carbon monoxide; FCT = fibrous connective tissue.

### Preparation of samples

#### Drug testing

All the deceased underwent drug testing using two different, validated methods. The first, a screening method used to detect traces of drugs, was an enzyme-multiplied immunoassay technique (EMIT) (Abbott Diagnostics, Chicago, IL, USA). To confirm these results, combined GC–MS was performed as a second screening method. The same application was used for substance quantification, as demonstrated by Vycudilik 27. GC–MS has very high sensitivity and specificity, which permits the detection of morphine in different bodily fluids such as blood, urine and bile, as well as tissues such as the liver and the brain. Gas chromatography was carried out in combination with a mass spectrometer, MAT 112® (Spectromat Massenspektrometer GmbH, Bremen, Germany). The GC–MS data were analysed using the Integrator 3390A by Hewlett Packard® and a LABCOM.1 computer (MSS-G.G.; Hewlett-Packard, Böblingen, Germany). A comparison of the specimens' peak areas with known standard concentrations yielded quantitative results 27.

As defined by Goodman & Gilman, heroin-related death is usually the result of respiratory failure 28. Consequently, the concentration of morphine was determined in the medulla oblongata, the cerebellum and, if available, in the blood and urine. Blood and urine samples were tested for 6-monoacetylamine (6-MAM) and other psychoactive substances (e.g. cannabis, benzodiazepine, caffeine, etc.). Polydrug use was assumed when more than one drug, including alcohol, was detected. Deaths as a consequence of heroin intoxication alone were classified as pure heroin-related deaths, whereas deaths due to heroin use in combination with other drugs and/or alcohol were classified as polydrug heroin-related deaths.

#### Histological and quantitative morphometric analysis

**Figure 1:**
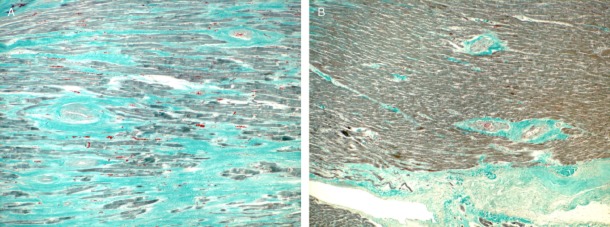
Sample pictures from myocardial specimen stained with Masson Goldner's Trichrome stain, each showing a myocardial histological specimen (×40 magnification) from a male (a) and female (b) drug-related death (DRD) case. Fibrous connective tissue (FCT) can be identified clearly by light-green colour

**Figure 2:**
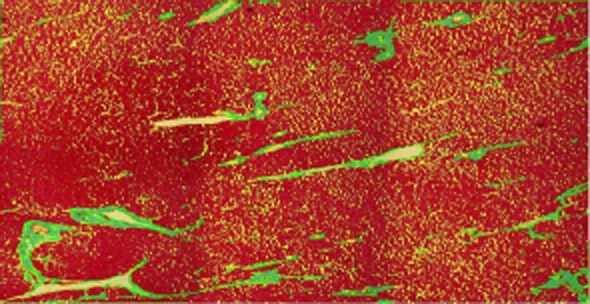
A sample picture from the morphometric LUCIA 1.16.2 software, showing a myocardial histological specimen (×40 magnification), as well as the amount of the different types of tissue that could be identified. Cardiac tissue (red: 87.98%), fibrous connective tissue (FCT) (green: 4.54%), artefacts (yellow: 7.49%)

To conduct a histological analysis, the standard paraffin-embedding procedure was applied to a total of 99 myocardial specimens. In addition to pre-set haematoxylin and eosin (H&E) dyes, Masson trichrome stains were performed to allow a better visualization of FCT (Fig. 1a,b). Subsequently, specimens were viewed under a ×40 magnification and recorded with a digital camera (DN100, Nikon®; Nikon GmbH, Vienna, Austria). Quantification of FCT in the specimens was permitted by the morphometric software LUCIA Net version 1.16.2^©^ (Laboratory Imaging, Praha, Czech Republic), Laboratory Imaging using NIS Elements 3.0® (Laboratory Imaging). The different colour shades were determined using cross-hairs and defined as follows: ‘red’ (corresponding to myocardial tissue), ‘green’ (corresponding to FCT) and ‘yellow’ (corresponding to artefacts) (Fig. 2). Accordingly, the amount of the colour ranges in each specimen sample was calculated by means of LUCIA software.

#### Statistical evaluation

SAS® 29 and Microsoft Excel® for Microsoft Windows were used to conduct a statistical analysis. A general linear model (GLM) with the effects of opiate versus control groups and gender was applied. As the residuals from this analysis were not distributed normally (*P* = 0.0001, using the Shapiro–Wilks statistic), data were log-transformed (*P* = 0.6651). Results were significant at *P* < 0.05.

## Results

### Drug testing

Of all the 76 observed drug-related deaths (DRD) (1993–94) included in this study, four autopsy files were not available (Table 2). Therefore, clinical files from the hospital were studied regarding drug consumption and health status of those deceased instead of the autopsy files. Almost all (93.42%) the victims showed fresh needle punctures, whereas to a greater or lesser extent 65.79% of them exhibited old scars from repeated punctures. More than two-thirds (67.11%) were polydrug opiate users: the same proportion could be classified as heroin addicts (6-MAM). The existence of codeine was proved in more than three-quarters of cases (76.32%) whereas, as far as additionally detected (psychoactive) substances were concerned, nicotine (cotinine) was the leading substance with 35.53%, followed by cannabis (32.89%), benzodiazepine (22.37%), caffeine (9.21%), cocaine and tricyclic antidepressants (TCA) (each 2.63%) and others (propyphenanzone, theophylline, barbiturate—each 1.32%).

**Table 2:** Overview of additionally detected substances in the opiate groups

Drug-related deaths	1993	1994	Total
	30	46	76
Missing autopsy files (clinical files only)		4	4
ELISA test only	1		1
Morphine level in blood or liver, bile only (no CNS)		2	2
Polydrug use (except of alcohol)	16	20	36
Polydrug use (only plus blood alcohol > 0.29 ng/g)	6	12	18
Polydrug use (inclusive of alcohol)	21	30	51
Scars (old punctures)	26	25	50
Fresh punctures	31	40	71
Codeine	24	34	58
Methadone	2	6	8
6-Monoacetylcodeine	1		1
Dihydrocodeine		1	1
6-Monoacetylmorphine (6-MAM)	23	28	51
Additional detected substances			
Nicotine, cotinine	9	18	27
Cannabis	11	14	25
Benzodiazepine	7	10	17
Caffeine	4	3	7
Benzoylecgonine (cocaine)		2	2
Amitriptyline [tricyclic antidepressants (TCA)]	1		1
Doxepine (TCA)	1		1
Propyphenanzone	1		1
Theophylline		1	1
Barbiturate		1	1

CNS = central nervous system; ELISA = enzyme-linked immunosorbent assay.

Morphine concentration in the central nervous system (CNS) was 4–2400 ng/g (mean 389.03 ± 420.05 ng/g) in the cerebellum and 5–1600 ng/g (275.52 ± 240.99 ng/g) in the medulla oblongata, respectively.

### Morphological results

**Figure 3:**
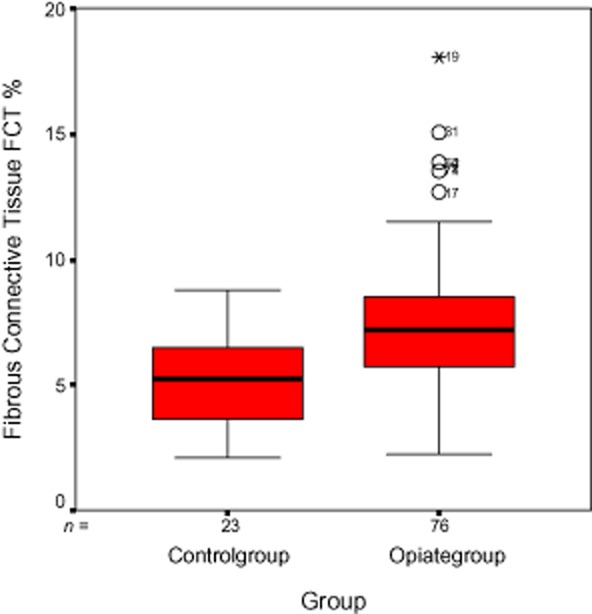
Box-plot graph presenting the distribution of fibrous connective tissue (FCT) in the myocardium of the control versus opiate group. The results show a significant difference between the control and the opiate group (*P* = 0.0012)

All 99 hearts of both groups exhibited physiological size. The mean heart weight was 282 g in the control and 293 g in the opiate group. A summary of the features regarding age and gender distribution as well as causes of death of both groups reviewed is provided in Table 1. The statistically significant differences between the control and the opiate group with respect to the quantity of myocardial fibrosis are shown in Fig. 3.

In the control group, in particular, the mean amount of connective tissue was 5.2 ± 1.7% (females: 4.45 ± 1.23%; males: 5.50 ± 1.78%). The percentage of connective tissue in this group ranged from 2.1 to 8.8%, which was still physiological for this age group. In contrast, the mean amount of FCT in the opiate group was 7.6 ± 2.9% (females: 6.30 ± 2.19%; males: 7.91 ± 3.01%). The percentage of FCT ranged from 2.2 to 18.1%, which clearly indicated fibromuscular dysplasia.

**Figure 4:**
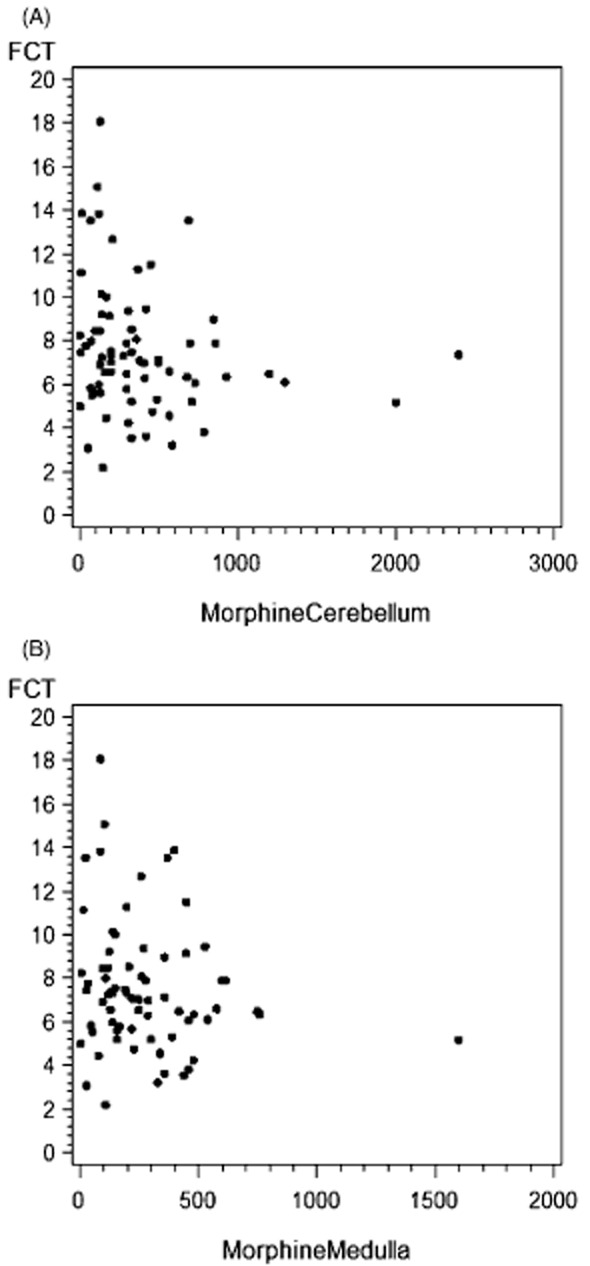
Presentation of Pearson's correlation of fibrous connective tissue (FCT) and morphine concentration in the central nervous system (CNS). Both graphs (a, cerebellum; b, medulla oblongata) demonstrate that there is a negative bias between FCT in the myocardium and morphine concentration in the cerebellum (*r* = −0.19032, *P* = 0.1119) and the medulla oblongata (*r* = −0.17117, *P* = 0.1535), respectively

These results revealed that there was a significant difference in the amount of FCT between the opiate group and the control group (*P* = 0.0012), and a significant difference in the amount of FCT between females and males (*P* = 0.0383). The effect of the interaction of opiate versus control and gender was tested and found to be insignificant (*P* = 0.8116). An additional analysis revealed that there was no significant interaction of age and FCT in the myocardium (*P* = 0.8472). There was no significant difference (*P* = 0.2349) between the tested groups with regard to the proportion of myocardial tissue. In order to determine whether there is a correlation between morphine concentration in the CNS and FCT in cardiac tissue, we introduced Pearson's correlation to measure the strength of the questioned relationship (Fig. 4a,b).

## Discussion

The findings presented in this retrospective study indicate clearly that there is a strong correlation between long-term opioid-accentuated drug abuse and fibrotic alterations in the myocardium.

It is well known that opiate abuse has a high incidence of cardiac complications, as shown by Burke *et al*. 30, Dettmeyer *et al*. 14, Stringer *et al*. 10 and Nerantzis *et al*. 31. Initially, the acute effects of heroin on the heart were first explored by Brashear *et al*. using dogs and included hypotension and bradycardia, which were caused by peripheral vasodilatation, reduced peripheral resistance and the release of histamine as well as the inhibition of baroreceptor reflexes 32. From a clinical viewpoint, opioids, especially heroin, may cause arrhythmias and pulmonary oedema as well as reducing cardiac output, as presented by Frishman *et al*. in 2003 33.

This study provides further evidence that chronic opioid abuse may be a risk factor contributing to the development of fibrotic remodelling in the heart during and even after opiate abuse. Our findings are in line with the results of previous histopathological studies by Passarino *et al*. and Milroy & Parai 13,34. Different quantitative methods are available to determine the extent of myocardial fibrosis: the point-counting method, computer image analysis and the biochemical method for quantifying myocardial scarring are some examples. However, according to Kitamura *et al*., these measurement methods show high intra-observer variability 35.

Distinct biopsy locations inside the heart may also lead to different results concerning the amount of myocardial fibrosis, as described in other publications (Table 3). As a consequence, it can be concluded that the mean percentage of fibrotic tissue depends upon several factors, such as anatomic location, method and observer, and ranges from 0.4 to 10% or more.

**Table 3:** Overview of several publications describing physiological proportion of fibrous connective tissue (FCT) in the myocardium

Physiological FCT in myocardium %	Published by	Year of publication	Reference number
0.4–1.0	St John Sutton *et al*.	1980	36
1.1 ± 0.5	Tanaka *et al*.	1986	37
1.95 ± 0.07	Querejeta *et al*.	2000	38
2–10	Vasiljević *et al*.	2001	39
6.8 ± 2.2	Gaspard & Pasumarthi	2008	40

In the study presented here, the control group had a mean value of FCT of 5.2%. Similar values had been found previously by Gaspard & Pasumarthi, where the mean value was 6.8 ± 2.2% 40.

According to studies conducted by Vasan *et al*. 41 and Campbell *et al*. 42, both describing gender-related differences in the cardiac structures, women have less interstitial fibrosis than men of the same age. This matches our results, which reveal a significant difference between females and males in both groups (*P* = 0.0103). Owing to the somewhat narrow age range of both groups from teenagers to young adults (opiate group: 15–38 years; control group: 15–41 years), a significant interaction of the variables age and FCT in the myocardium is not indicated (*P* = 0.8472).

In our study, almost all (93.42%) the deceased showed fresh needle punctures, implying extensive intravenous opiate abuse, while almost two-thirds (65.79%) suggested chronic intravenous application due to distinct scars. The fact that more than two-thirds of the victims were polydrug opiate abusers also underscores the cumulative effect of psychoactive and CNS depressant substances, amplifying the harmful consequences of opioids and also interfering with heart tissue. The morphine concentration in the CNS are levels measured at the time of death and therefore do not represent the period of opiate consumption as, for example, the scars. Due to the very low existence of stimulating drugs such as cocaine (only 2.63%), in our study the impact of their FCT triggering effect on the hearts could be excluded.

Furthermore, as has been the case with recent findings by Nerantzis *et al*. and Passarino *et al*. 31,34, this study clearly supports the theory that not only does the chronic abuse of cocaine and/or amphetamines cause fibrotic remodelling in the heart, as demonstrated by Milroy & Parai in 2011 13, but also that the long-term abuse of opioids harms cardiac tissue with similar effects, which may lead to the dysfunction or failures of electrical activity of the myocardium and different types of arrhythmia, such as Q-T interval prolongation and torsades de pointes (TdP), as shown by Stringer *et al*. in 2009 10.

However, the path leading to the development of myocardial fibrosis after chronic opiate abuse can instead be theorized as follows: recurrent opioid abuse, especially the intravenous injection of heroin, causes a decrease in the respiratory rate through a lessened sensitivity of neurones in the respiratory centre located in the brain stem. This depressed respiratory drive eventually causes hypoxia, also in the heart tissue, by hampering the initialization of oxygen. This critical under-supply of oxygen in the myocardium induces, in turn, apoptosis in myocytes, in almost the same manner as can be observed in a myocardial infarction. This pathogenesis then disembogues into stimulation of cardiac remodelling, which results ultimately in a fibrosis.

Therefore, the negative tendency between morphine level in the CNS and FCT in the cardiac tissue in our study suggests that chronic opioid users (mainly older, more experienced individuals) may need a lower concentration of morphine to reach an overdose due to the long-term effect of opioids on cardiac tissue compared to less experienced, younger addicts.

In conclusion, although the results of this work indicate a significant correlation between long-term opioid-accentuated drug abuse and myocardial fibrosis, it must be kept in mind that the study presented here still has some limitations: first, as a retrospective limitation, analysed heart tissues originated from samples taken exclusively for forensic purposes; and secondly, no hair samples were taken and analysed in order to evaluate the continuance and to determine the type of additional drugs used potentially in the time preceding death. Thus, case files, forensic autopsy documentations and police reports, all studied meticulously, served solely as information regarding previous drug habits. However, all the cases used in this study were selected carefully from this specific period in the 1990s (1993–94), and the co-consumption of opioids and stimulant drugs such as cocaine could be excluded, on one hand, for empirical reasons, as stated by Risser *et al*. 8, and on the other hand could be verified clearly in our study (2.63%). Thirdly, in our study population, pure heroin-related death represented only about a third of the heroin-related deaths: in the other two-thirds, morphine derivatives in combination with another drug and/or alcohol could be detected. In this context, however, it must be kept in mind that the co-consumption of other CNS depressant or psychoactive drugs can increase substantially the feasibility of a fatal outcome following injection of heroin as a consequence of the amplification effects of heroin. This fact was also described in the 2009 report by the EMCDDA 43: the negative cardiovascular effects of cocaine are amplified when it is co-administered with opioids. Animal studies also indicate that cocaine, as well as opioids, can induce respiratory depression, which could increase overdose risk. In addition, cocaine can initially mask the sedative effects of opioids, thereby increasing the risk of a later overdose. Opioids, benzodiazepines and alcohol are all depressants of the central nervous system. Their concomitant use can lead to marked respiratory depression, resulting in a high risk of fatal and non-fatal overdoses. Older drug users may also face delayed metabolism of benzodiazepines and there is an increased danger of respiratory depression when used with methadone 43. Fourthly, the quality of heroin was not asserted in detail although the codeine detected in about three-quarters of the victims indicate a certain heroin impurity, as already described by Risser *et al*. 8. However, in an earlier study by Risser *et al*. 7, the results did not substantiate the widely held belief that increases in heroin-related deaths could be explained by an increase in the quality of heroin.

None the less, this primarily descriptive survey indicates the need for additional examinations and further prospective case–control studies in order to determine the actual role played by opioids in the development of fibrosis, especially in the myocardium, and the contribution of such opiates to the functional impairment of the heart's circulation system.

Finally, in accordance with both our recent findings and recent studies by George *et al*. 44 and Fareed *et al*. 45, it is urgently recommended that patients receiving substitution therapy with morphine (such as methadone) 9 should be monitored closely and that they undergo regular cardiological examinations in order to receive the appropriate treatment and prevent advanced destruction of the cardiac tissue.
